# The Effect of Preeclampsia on the Cell Adhesion, Migration, and Proliferation Potential of Human Umbilical Cord-Derived Mesenchymal Stem Cells

**DOI:** 10.7759/cureus.100783

**Published:** 2026-01-04

**Authors:** Nabila Rasheed, Nisha Zahid, Syeda Saima Razzaq, Anum Siraj, Kanwal Haneef, Arhum Mustajab, Jasmeet Kaur

**Affiliations:** 1 Pharmacology and Therapeutics, Sapienza University of Rome, Rome, ITA; 2 Neuropharmacology, Instituto de Investigación Biomédica de Málaga y Plataforma en Nanomedicina (IBIMA Plataforma BIONAND), Malaga, ESP; 3 Pharmacology and Toxicology, Sapienza University of Rome, Rome, ITA; 4 Dr. Zafar H. Zaidi Center For Proteomics, University of Karachi, Karachi, PAK; 5 Internal Medicine, University Hospitals of Derby and Burton NHS Foundation Trust, Derbyshire, GBR; 6 Medicine, Sapienza University of Rome, Rome, ITA

**Keywords:** cell cycle, cord tissue, mesenchymal stem cells, preeclampsia, wound healing

## Abstract

Background: Human umbilical cord-derived mesenchymal stem cells (hUC-MSCs) are a well-known source in the field of regenerative medicine. However, their biological features and perinatal environment are compromised by various conditions, such as preeclampsia. Pregnancy-related complications lead to functional impairments, making the cord-isolated mesenchymal stem cells (MSCs) less efficient in self-renewal and regenerative capacity. Therefore, the purpose of this study was to assess the adhesion, migration, and proliferation potential of hUC-MSCs in preeclamptic conditions.

Methods: MSCs were obtained from umbilical cords of normal and preeclamptic pregnancies, with three samples collected per group. The functional properties of hUC-MSCs, including adhesion, proliferation, migration, and colony formation, were assessed through cell adhesion, wound-healing, and colony-forming unit assays. Moreover, the proliferative potential of hUC-MSCs was determined by calculating the number of population doublings (NPD) and population doubling times (PDT). The expression level of cell cycle-related genes is examined by quantitative PCR.

Results: The MSCs isolated from preeclamptic cords exhibit higher PDT (p < 0.01) and reduced NPD (p < 0.05), adhesion (p < 0.001), proliferation (p < 0.01), wound-healing (p < 0.01), and colony formation capabilities. Additionally, preeclampsia-affected hUC-MSCs showed significantly downregulated cell cycle genes, including CDCA2 (0.86-fold, p < 0.01), CDCA8 (0.049-fold, p < 0.001), CDC20 (0.34-fold, p < 0.01), and CCNA2 (0.25-fold, p < 0.01).

Conclusion: The study concludes that MSCs derived from preeclampsia cord show decreased adhesion, migration, and proliferation capabilities compared to normal hUC-MSCs. These findings show that pregnancy complications can influence the biological properties of perinatal stem cells. However, further research is necessary to investigate the underlying mechanisms via which preeclampsia affects the proliferative potential of stem cells, and to identify strategies to increase the proliferation rates of these cells.

## Introduction

Bone marrow, amniotic fluid, dental pulp, umbilical cord blood, Wharton’s jelly, cord, and adipose tissue are rich sources of mesenchymal stem cells (MSCs) or myofibroblast-like cells [1]. The capability of MSCs to self-renew and specialize into several cell lineages is one of their most unique characteristics [2]. Numerous clinical trials are being conducted to examine the effectiveness of MSCs in treating a range of conditions, such as full-thickness burns, hearing loss, stroke, autism, Alzheimer's disease, cerebral palsy, bronchopulmonary dysplasia, osteoarthritis, cartilage damage, and inborn metabolic disorders such as diabetes and obesity [3,4].

The most recognized type of stem cells is human umbilical cord-derived mesenchymal stem cells (hUC-MSCs). They exhibit the ability to regenerate and differentiate into several lineages, and they are less immunogenic. They are desirable candidates for autologous or allogeneic therapy in the treatment of various illnesses due to their ability to differentiate into the three germ layers [5]. As compared to other stem cell sources, hUC-MSCs have various benefits, such as simpler isolation, fewer requirements for human leukocyte antigen (HLA) matching, a lower risk of graft-versus-host disease (GVHD), and improved accessibility for transplantation [4,6]. Their use in tissue engineering and gene therapy has been exhibited in different studies. However, therapeutic uses of hUC-MSCs in tissue repair and regenerative medicine could possibly be severely compromised by various metabolic disorders [7,8]. It has been reported that changes in p16Ink4a function caused by specific metabolic disorders during pregnancy, such as gestational diabetes, have been demonstrated to inhibit cyclin-dependent kinase, which arrests the cell cycle, and lower MSC proliferation [9,10].

Preeclampsia (PE) is recognized as a placental disorder and a leading cause of mortality among pregnant women and their fetuses. This morbid pregnancy condition affects 2%-8% of pregnancies worldwide. Preeclampsia may cause an early birth or termination of the pregnancy. Preeclamptic mothers have been presented to have a lower number of circulating endothelial progenitor cells when compared to controls [11]. According to research, endothelial damage and dysfunction are caused by reactive oxygen species (ROS) production in preeclampsia. Elevated ROS can damage DNA and proteins, leading to reduced proliferation and increased senescence of hUC-MSCs. Pro-inflammatory cytokines such as tumor necrosis factor-alpha (TNF-α) and IL-6 activate nuclear factor-kappa B (NF-κB) signaling, which suppresses regenerative pathways and promotes apoptosis. Moreover, endothelial dysfunction decreases nitric oxide (NO) bioavailability and alters vascular endothelial growth factor (VEGF) signaling, compromising the microenvironment required for normal stem cell adhesion and migration. Collectively, these molecular disturbances impair the regenerative capacity of hUC-MSCs derived from preeclamptic pregnancies [6,9]. Literature reports that preeclampsia during pregnancy adversely affects the transcriptomic profile and cellular functions of umbilical cord-derived MSCs, which can disrupt the expression of key genes that regulate cell cycle, adhesion, migration, and proliferation. These molecular changes lead to functional impairments, making the cells less efficient in self-renewal, tissue homing, and regenerative capacity [12]. However, the consequences of preeclampsia on the adhesion, migration, and proliferation of hUC-MSCs are still unknown.

hUC-MSCs are highly valuable for regenerative medicine because they are easily accessible, ethically acceptable, proliferate rapidly, and have strong immunomodulatory and tissue-repair capabilities. If preeclampsia affects these core properties, it could impact the quality and clinical success of hUC-MSCs stored in stem cell banks. Therefore, this study aims to assess how preeclampsia impacts MSCs’ ability to adhere, migrate, and proliferate. It will expand our understanding of using hUC-MSCs in regenerative medicine and stem cell banking.

## Materials and methods

Collection of human umbilical cord tissue

This study was conducted at Dr. Zafar H. Zaidi Center for Proteomics, with approval from the University of Karachi’s ethical committee (Approval No: IBC KU-125/2020). Umbilical cords were collected from full-term cesarean deliveries of normal and preeclamptic mothers (diagnosed according to the American College of Obstetrics and Gynecology (ACOG) 2020 criteria: blood pressure ≥140/90 mmHg after 20 weeks), after taking informed consent. All mothers with a history of problematic pregnancies with fetal anomalies or intrauterine growth restriction, gestational diabetes, ruptured membranes, placenta previa, pregnancy-induced hypertension, or patients who were positively tested for hepatitis were excluded from this research.

Isolation and expansion of hUC-MSCs

To ensure consistency, approximately 10 cm segments of umbilical cord (n = 3) were collected from preeclampsia and healthy pregnant women. Umbilical cord tissue was washed in a sterile phosphate-buffered saline (PBS) solution to remove any remaining blood. After that, the cord tissue was cut into 3 mm pieces and partially digested with trypsin for 30 minutes at 37°C. The trypsin-digested 5-6 cord tissues were cultured at 37°C with 5% CO2 in Dulbecco's modified Eagle medium (DMEM) supplemented with 10% fetal bovine serum, sodium pyruvate, Pen/Strep, and L-glutamine. After 70-80% cell confluency, the cells were washed with sterile PBS, and then 2-3 mL of 0.25% trypsin was added to the cells, and the flasks were incubated for four to five minutes at 37°C. After the incubation period, the trypsin reaction was stopped by adding complete DMEM. The cell suspension was transferred into a 15 mL Falcon tube and centrifuged at 300 x g for eight minutes. Finally, cells were seeded into tissue culture flasks [13]. Passages 1-2 cells were used throughout the study.

Morphological observation of hUC-MSCs

The morphological changes of the cells isolated from the cords of the normal and preeclamptic patients were examined using an inverted microscope (CKX41, Olympus, Tokyo, Japan) at a 10x magnification. The characterization of MSCs has been conducted and documented elsewhere in previous studies [14].

Colony-forming unit assay

MSCs were cultured at a density of 1500 cells per cm^2^ in a cell culture flask for one week. After the fixation of the cells with paraformaldehyde (PFA), the cells were incubated with 0.5% crystal violet. Colonies were washed with distilled water, and images were captured under a microscope (CKX41, Olympus).

Cell adhesion assay

For the cell adhesion assay, 1500 cells per cm² from the preeclampsia and control groups were seeded in a cell culture flask. The cells were then observed at 0 minutes (shortly after culturing), 30 minutes, one hour, and two hours. The following formula was used to evaluate cell adhesion:

% Percentage = No. of cells adhered to the flask surface/No. of floating cells in media.

Wound healing potential

To assess the wound closure capacity of control and preeclampsia hUC-MSCs, an in vitro scratch assay was performed. Cells were cultured in a T-25 flask, and a layer of confluent cells was scratched with a 100 µL sterile tip. Under a phase contrast microscope, cell migration was seen at six hours, 24 hours, and 48 hours (CKX41, Olympus).

The following formula was used to determine the percent of wound healing potential:

% of wound closure = $\left[\frac{h_i - \Delta h}{h_i}\right] \times 100\%$

Where *hi* is the area of the wound measured immediately after scratching, and *Δh* is the wound area measured at six, 24, and 48 hours after scratching.

MTT assay

The cell cytotoxicity assay was conducted according to the previously described method by Rashid et al. (2022) [15]. The 3 × 104 cells were plated in a well of a 96-well plate and allowed to attach to the surface for 24 hours. After the removal of media, 5 mg/mL MTT (3-(4,5-dimethylthiazol-2-yl)-2,5-diphenyltetrazolium bromide) was added for four hours, and then 100 µL of dimethyl sulfoxide was added for 30 minutes. Absorbance was measured using a microplate reader (Beckman Coulter, Brea, CA) at 570 nm wavelength.

Population doubling time

To calculate the population doubling time (PDT) and the time needed for the cells to reach their 80-90% confluency, normal and preeclamptic cells were seeded at 10,000 cells per well on 24-well culture plates. For the calculation of PDT, the following formula was used:

PDT = culture time (CT)/population doubling number (PDN)

$\text{Population doubling number} = \log\left(\frac{N_i}{N_f}\right) \times 3.31$

Where Ni = initiating numbers; Nf = numbers at harvesting stage.

Cell cycle-related gene analysis by quantitative PCR

The RNA was isolated using the optimized TRIzol protocol. The cell pellet was resuspended in TRIzol reagent, and then chloroform was added to facilitate the phase separation. For RNA isolation, the top aqueous layer was carefully removed after centrifuging the mixture at 12,000 × g. To precipitate the RNA, cold isopropanol was added, and the mixture was centrifuged at 10,000 × g for 10 minutes at 4°C. In the next step, 75% ethanol was added to the pellet obtained after centrifugation, allowed to air dry, and then suspended in nuclease-free water. A spectrophotometer was used to measure the total yield of RNA. The reverse aid first strand complementary DNA (cDNA) synthesis kit (Invitrogen, Waltham, MA) was used to reverse transcribe the RNA into cDNA. Briefly, a reaction mixture including deoxynucleotide triphosphates (dNTPs), RNase inhibitors, reverse transcriptase, oligo (dT) primers, reaction buffer, and nuclease-free water was incubated with 1 µg of RNA for one hour at 42°C. Using quantitative PCR (qPCR), the expression levels of cell cycle-specific genes were evaluated. The cDNA from control and preeclamptic hUC-MSCs was amplified in the SYBR green master mix (Master Mix-R, ABM) using a real-time PCR instrument (Applied Biosystems, Waltham, MA). Table 1 lists all of the primer sequences utilized for qPCR.

**Table 1:** List of cell cycle primers.

Gene	Primer sequence (5’-3’)		Annealing temperature	Accession number
	Forward	Reverse		
CDCA2	TTGCGTAAAGGAGGAACACC	CAAGATTCTCCCCCTTGTCA	60°C	NM_001317907.1
CDCA8	CAGACAGGCAGAACCTCCTC	CAAGGGCGAAGTAGTCAAGC	60°C	NM_001256875.2
CDC20	TATGGCGCTGTTTTGAGTTG	CTGAGGTGATGGGTTGGTCT	60°C	NM_001255.3
CCNA2	CCTGCAAACTGCAAAGTTGA	AAAGGCAGCTCCAGCAATAA	60°C	NM_001237.5
GAPDH	GCTCTCTGCTCCTCCTGTTC	CCATGGTGTCTGAGCGATGT	60°C	NM_002046

The cycle threshold (CT) values were recorded, and the 2^−ΔΔCT method was used to calculate the fold change for gene expression, using GAPDH as the housekeeping gene.

ΔCt = Ct (Gene of interest) - Ct (Housekeeping gene)

ΔΔCt = ΔCt (Sample) - ΔCt (Control)

Fold change = 2^ (- ΔΔCt).

Statistical analysis

SPSS version 20 software (IBM Corp., Armonk, NY) was utilized to analyze the data. All values are presented as mean +/- SEM. The t-test was used to determine both groups' mean and SEM. A P-value of <0.05 was used to determine statistical significance between the groups.

## Results

Cell morphology and colony-forming unit assay

**Figure 1:**
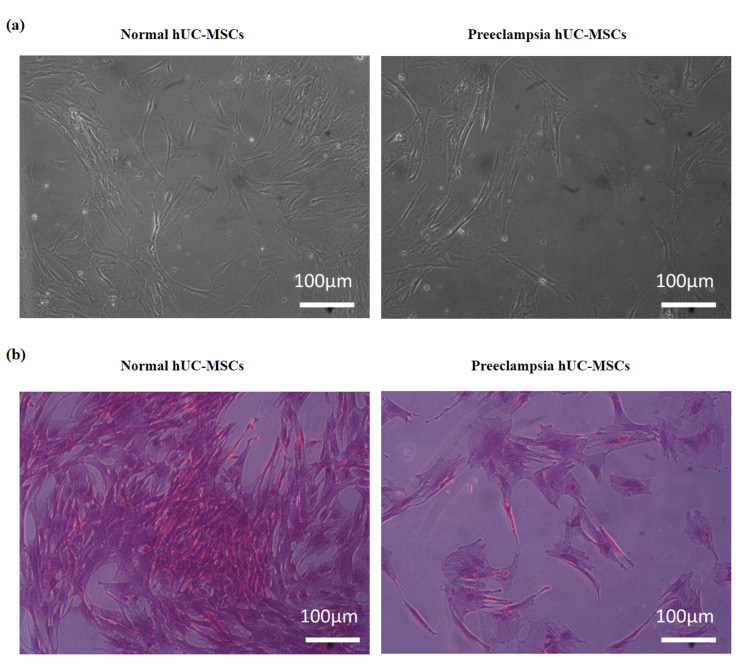
Morphological representation of normal and preeclampsia hUC-MSCs. (a) Normal and preeclampsia hUC-MSCs exhibited a fibroblast-like morphology. (b) Preeclampsia hUC-MSCs exhibited fewer colonies compared to normal cells. Images were taken at 10x magnification under phase contrast microscopes. hUC-MSCs: human umbilical cord-derived mesenchymal stem cells.

At P1, control and preeclampsia hUC-MSCs exhibited a fibroblast-like morphology under a phase contrast microscope (Figure 1A). The colony-forming unit (CFU) assay was employed to examine the presence of mesenchymal progenitors. Following one week of cell seeding, purple-colored cell colonies of hUC-MSCs were observed. The preeclampsia group showed fewer colonies (10 ± 2) than the control group (33 ± 4) (Figure 1B).

Adhesion profiling

**Figure 2:**
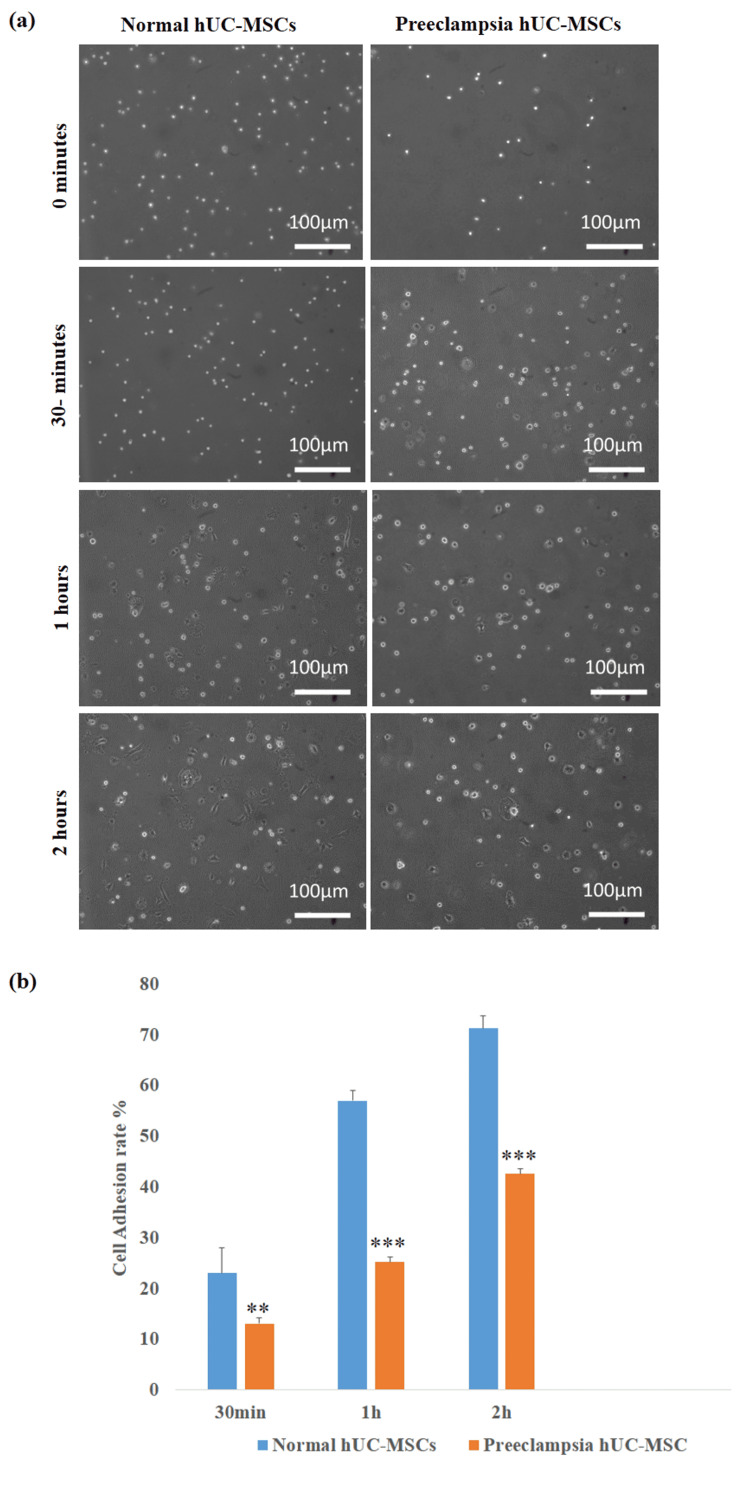
Effect of preeclampsia on adhesion ability of human umbilical cord-derived mesenchymal stem cells. (a) Images of the control preeclampsia group hUC-MSCs at 0 minutes, 30 minutes, one hour, and two hours of cell seeding. (b) Bar diagrams representing the cell adhesion rate of normal and preeclampsia hUC-MSCs. The data represent ± standard error of means (SEM) of three independent experiments (n = 3). * represents statistically significant value with reference to the control (**P < 0.01, *** P < 0.001). hUC-MSCs: human umbilical cord-derived mesenchymal stem cells.

To determine the cell adhesion potential of control and preeclamptic MSCs, an in vitro adhesion assay was conducted. As shown in Figure 2, a significant decrease in the cell adhesion rate at 30 minutes (**P < 0.01), one hour (***P < 0.001), and two hours (***P < 0.001) was observed in the preeclampsia group.

Cell proliferation assay

**Figure 3:**
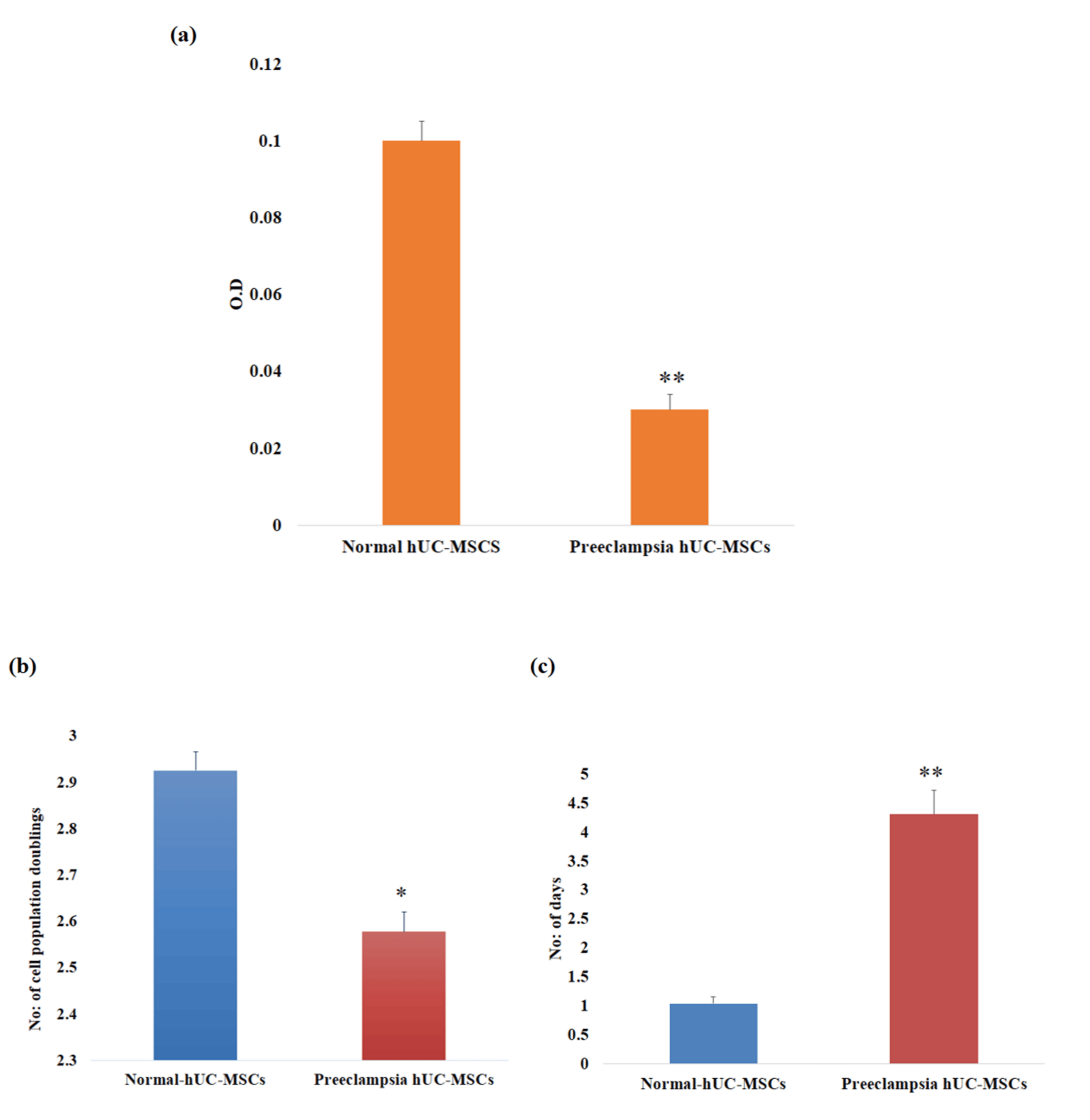
Cell proliferation analysis of human umbilical cord-derived mesenchymal stem cells. (a) The MTT assay indicates a significant decrease in the number of cells in preeclampsia hUC-MSCs. (b) Preeclampsia hUC-MSCs showed significantly decreased NPD as compared to the control group. (c) The PDT was significantly higher in the preeclampsia hUC-MSCs group compared to the control group. The data represent ± standard error of means (SEM) of three independent experiments (n = 3). * represents statistically significant value with reference to the control (*P < 0.05, **P < 0.01). hUC-MSCs: human umbilical cord-derived mesenchymal stem cells; MTT: 3-(4,5-dimethylthiazol-2-yl)-2,5-diphenyltetrazolium bromide; NPD: number of population doublings; PDT: population doubling time.

For proliferation analysis, fluorescence intensity was measured for preeclampsia and control hUC-MSCs at the corresponding time point following incubation with MTT dye for four hours. The preeclampsia hUC-MSCs showed a significant reduction (**P < 0.01) in cell proliferation (Figure 3A). These results indicate that hUC-MSCs isolated from preeclamptic cords were less metabolically active. The analysis of population doubling time (PDT) and number of population doublings (NPD) provided additional evidence of cell proliferation. The preeclamptic hUC-MSCs group exhibited significantly lower NPD (*P < 0.05) than the control group, but PDT increased significantly (**P < 0.01) in the preeclamptic hUC-MSCs (Figures 3B, 3C).

Wound healing potential and transcriptional changes in preeclamptic cord-derived hUC-MSCs

**Figure 4:**
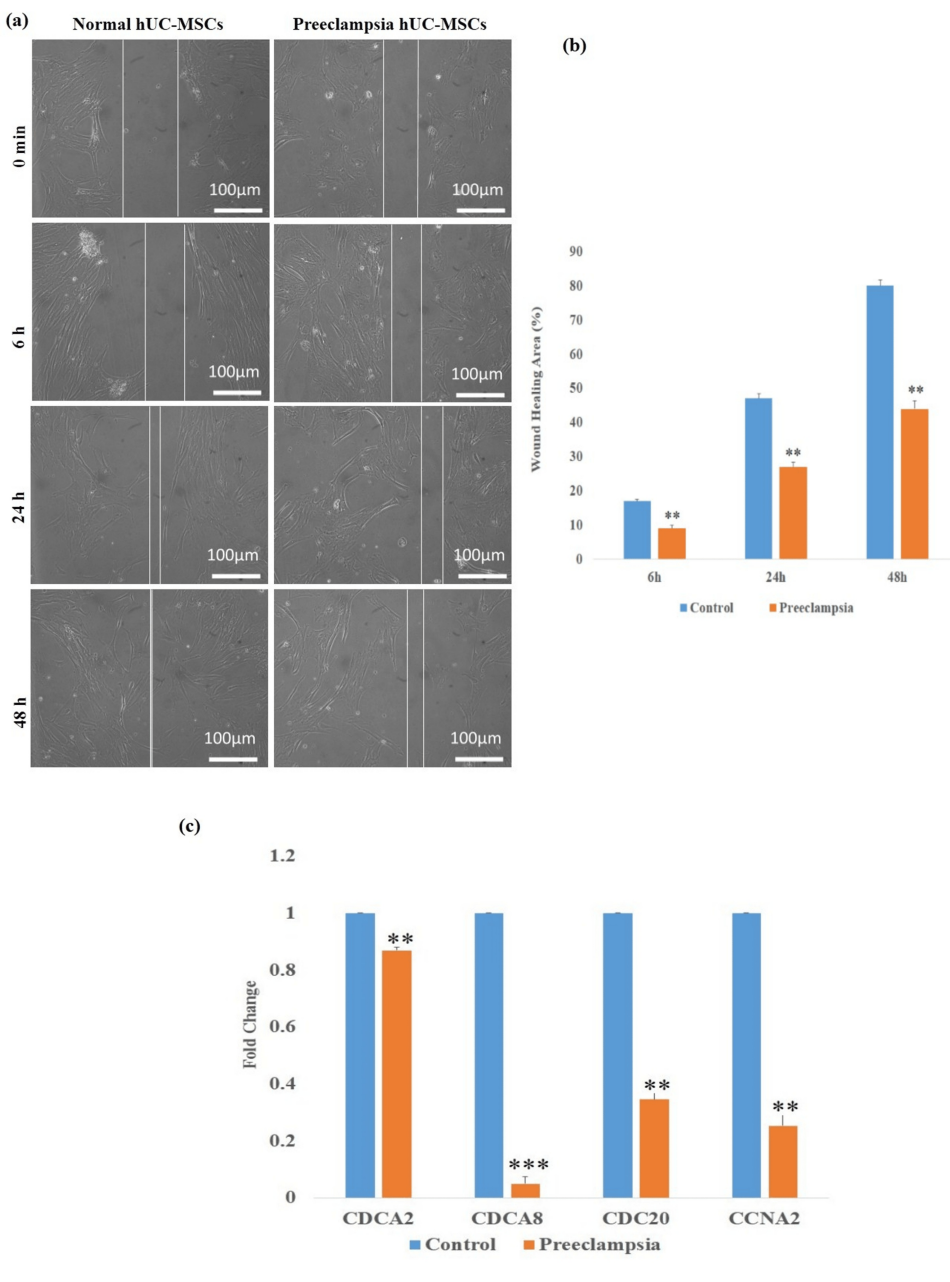
Effect of preeclampsia on wound healing potential and cell cycle-related genes. (a) Images of hUC-MSCs from the control and preeclampsia groups at 0 minutes, six hours, 24 hours, and 48 hours after the scratch was given. (b) Bar diagrams representing the cell healing area of normal and preeclampsia hUC-MSCs. (c) Bar diagrams representing gene expression analysis of cell cycle genes CDCA2, CDCA8, CDC20, and CCNA2. The data represent ± standard error of means (SEM) of three independent experiments (n = 3). * represents statistically significant value with reference to the control (*P < 0.05, **P < 0.01, *** P < 0.001). hUC-MSCs: human umbilical cord-derived mesenchymal stem cells; GAPDH: glyceraldehyde 3 phosphate dehydrogenase; CDCA2: cell division cycle associated 2; CDCA8: cell division cycle associated 8; CDC20: cell division cycle 20 homologue; CCNA2: cyclin A2.

Normal and preeclamptic hUC-MSC wound-healing ability was assessed using an in vitro scratch assay. In both groups, the scratch's healing area was estimated at six, 24, and 48 hours. Preeclampsia hUC-MSCs showed decreased healing potential at six hours (**P < 0.01), 24 hours (***P < 0.01), and 48 hours (***P < 0.01) in comparison to control cells (Figures 4A, 4B). The percentages of wound-healing at various time points are presented in Table 2.

**Table 2:** Percentage (%) of wound-healing rate over time.

Groups	6 hours	24 hours	48 hours
Control	17%	47%	80%
Preeclampsia	9%	27%	44%

Also, we examined transcriptional changes between preeclampsia and untreated hUC-MSCs using fold-change calculation (2-ΔΔCt) of cell cycle genes. Preeclampsia hUC-MSCs showed significantly lower expression of proliferation genes, specifically CDCA2 (0.86-fold, P < 0.01), CDCA8 (0.049-fold, P < 0.001), CDC20 (0.34-fold, P < 0.01), and CCNA2 (0.25-fold, P < 0.01), in comparison to normal cells (Figure 4C).

## Discussion

The ability of hUC-MSCs to differentiate across many lineages makes them an effective tool in tissue regeneration research [14]. hUC-MSCs mainly deliver their therapeutic effects via promoting angiogenesis, cell division, and reducing apoptosis [4,15]. However, many metabolic conditions during pregnancy have been shown to have negative effects on hUC-MSCs' therapeutic potential [9].

Preeclampsia is a recognized placental condition that is one of the main causes of death for pregnant women and their fetuses [9,10]. Numerous clinical and laboratory studies have demonstrated that placenta-derived mediators, which are released in response to placental ischemia and reperfusion injury, are the initial cause of preeclampsia [16]. Preeclampsia during pregnancy adversely affects cellular and transcriptomic processes of umbilical cord-derived MSCs [17]. In the present study, we studied the effect of preeclampsia on the proliferative potential of hUC-MSCs.

Several studies have confirmed that the biological characteristics of fetal stem cells can be affected by the metabolic abnormalities that occur during pregnancy. It has been reported that MSCs obtained from human umbilical cords have the largest proportion of colony-producing abilities when compared to MSCs obtained from bone marrow, adipose, and other tissue sources [18,19]. According to our findings, the preeclampsia-affected women's hUC-MSCs displayed a larger spindle form and were less densely colonized compared to hUC-MSCs isolated from non-affected mothers. These results are consistent with past studies showing that metabolic problems during pregnancy impact MSCs' capacity to form colonies [19,20].

It has been documented in various studies that the rate of adhesion and migration of stem cells is significantly altered due to pregnancy-related metabolic alterations. The adhesion-specific genes, including integrin, vinculin, actin, ICAM1, and VEGFA, are reported to show decreased expression in these cells, which results in reduced cell adhesion and migration [21,22]. The cell adhesion assay result of the current study showed that preeclamptic cord-derived hUC-MSCs attached less frequently than control cells. Another essential requirement for the therapeutic application of hUC-MSCs is the evaluation of the rate of cell proliferation and survival. In this study, a low proliferation rate and a higher population doubling time were observed in preeclamptic cord-derived hUC-MSCs. These findings align with past research that shows the effect of metabolic diseases on the rates of stem cell growth [20,21].

The ability of the cells to multiply and go to the injured area, eventually resulting in wound closure, is known as wound healing potential. Numerous studies have demonstrated that metabolic illnesses gradually limit the potential for wound healing due to the changes in the hormone levels that interfere with normal healing processes [20,23]. Furthermore, it has been noted that high blood pressure and pregnancy-related hypertension obstruct cell migration and proliferation to the injury site [24]. In this research, we assessed the wound healing capacity of hUC-MSCs in preeclampsia and normal conditions. In comparison with normal cells, our results show a significant decrease in the healing potential of hUC-MSCs derived from mothers affected by preeclampsia. These findings strongly suggest that metabolic disorders of the donor are important variables to consider when using hUC-MSCs in clinical settings.

We performed cell cycle gene analysis to determine how preeclampsia affects the proliferation and division capabilities of MSCs. This impairment has been increasingly associated with the dysregulation of crucial cell cycle genes, active at different stages of cell division. Notably, genes such as CDCA2, CDCA8, CDC20, and CCNA2 show significantly reduced expression in preeclampsia-affected cells compared to healthy controls. CDCA2 encodes a regulatory subunit of protein phosphatase 1 (PP1), essential for targeting chromatin during mitotic exit, facilitating nuclear envelope reassembly, and DNA damage repair. Oxidative stress and inflammation, hallmarks of the preeclamptic placenta, contribute to the downregulation of CDCA2, resulting in delayed mitotic exit and compromised genome stability [25,26]. Similarly, CDCA8, a key component of the chromosomal passenger complex responsible for stabilizing chromatin-induced microtubules and spindle assembly, is downregulated in preeclamptic cells. This reduction destabilizes the mitotic spindle, causing chromosomal misalignment and activating spindle assembly checkpoints that delay the metaphase-anaphase transition [27]. CDC20, which activates the anaphase-promoting complex to trigger sister chromatid separation, also exhibits decreased expression in preeclampsia. This reduction impairs the timely progression into anaphase, leading to cell cycle arrest and further slowing cell proliferation [28]. Cyclin A2 (CCNA2) regulates both S phase progression and entry into mitosis by activating cyclin-dependent kinases, and is similarly repressed, diminishing CDK activity, impairing DNA replication, and delaying the G2/M transition. The molecular mechanisms underlying this downregulation are multifaceted. Elevated ROS in the preeclamptic placenta activates DNA damage response pathways such as ATM/ATR-p53, which transcriptionally repress cell cycle genes to induce cell cycle arrest. Moreover, hypermethylation of the promoter regions of CDCA8 and CDC20 genes further suppresses their expression. Additionally, increased pro-inflammatory cytokines, including TNF-α and IL-6, modulate NF-κB signaling pathways, which downregulate cyclins and cell cycle regulators. Impaired growth factor signaling diminishes PI3K/AKT and MAPK pathway activation, thereby weakening mitogenic signals essential for cell cycle progression.

Collectively, these molecular alterations lead to a delay in various cell cycle phases, particularly prophase, metaphase, and anaphase, culminating in longer population doubling times and slower trophoblast proliferation and migration [29,30]. This disruption of cell cycle dynamics not only affects placental growth but also impairs cytoskeletal remodeling necessary for effective trophoblast invasion into maternal tissues, a critical defect in preeclampsia pathogenesis. Thus, the decreased expression and function of cell cycle genes such as CDCA2, CDCA8, CDC20, and CCNA2 provide a mechanistic link between the cellular dysfunction observed in preeclampsia and the clinical manifestations of the disease. However, further investigation is necessary to fully understand the molecular mechanisms behind the decreased proliferation potential of preeclampsia cord-derived mesenchymal stem cells.

Limitations of the study

Despite the valuable insights generated, this study has several limitations. The relatively small sample size may limit the generalizability of the findings. The exclusive use of in vitro assays may not capture the complex in vivo environment in which hUC-MSCs naturally function. Although alterations in gene expression were identified, the underlying molecular mechanisms were not validated through functional studies. Future investigations using more samples, in vivo models, and mechanistic validation are essential. These steps will confirm these results and help define how preeclampsia affects the regenerative capacity of hUC-MSCs. Additionally, it will inform their suitability for clinical applications, including stem cell banking.

## Conclusions

To conclude, this study demonstrated that preeclamptic cells exhibited a higher doubling time as well as a decreased ability to adhere, migrate, and proliferate. Additionally, our results suggest that cell cycle genes may have a role in the reduced proliferation of hUC-MSCs. These findings show that pregnancy complications can influence the biological quality, therapeutic reliability, and long-term safety of stored perinatal stem cells. However, further investigation is required to fully determine the biological process and molecular dynamics behind the reduced adhesion, migration, and proliferation potential of preeclampsia-affected hUC-MSCs.
